# Supplementary material for: Phylogenetic Partitioning of Gansu Flora: Unveiling the Core Transitional Zone of Chinese Flora
Source: Plants (Basel). 2023 Aug 25;12(17):3060. doi: 10.3390/plants12173060 (PMC10490386; doi:10.3390/plants12173060)
Supplement: Supplementary file 1 [file plants-12-03060-s001.zip › Supplementary_Material.pdf]

# *Supplementary Material*

## 1 Supplementary Data

### 1.1 Regional Flora Books

References 95-104 in the main text.

### 1.2 Online Database

Catalogue of Life China. (2022). http://sp2000.org.cn/ [Accessed May 15, 2022]

Flora of China. http://www.iplant.cn/foc

Specimen records in the Chinese Virtual Herbarium (CVH). http://www.cvh.ac.cn/

### 1.3 References

References 105-184 in the main text.

### 1.4 Supplementary Data Matrix

The matrix format used to store and analyze data is the 'site-by-species' matrix. The site is a county-level geographical unit in our study. The sites are arranged in rows with the names of the rows being the names of the counties. The columns of matrix contain genera recorded in our species distribution data with the column names as the unique names of genus. The values in the matrix are binary (1/0) indicating the presence or absence of each genus in each county. Totally, our data matrix has 81 rows and 1,131 columns (see Supplementary_data_matrix.csv).

## 2 Supplementary Figures and Tables

### 2.1 Supplementary Figures

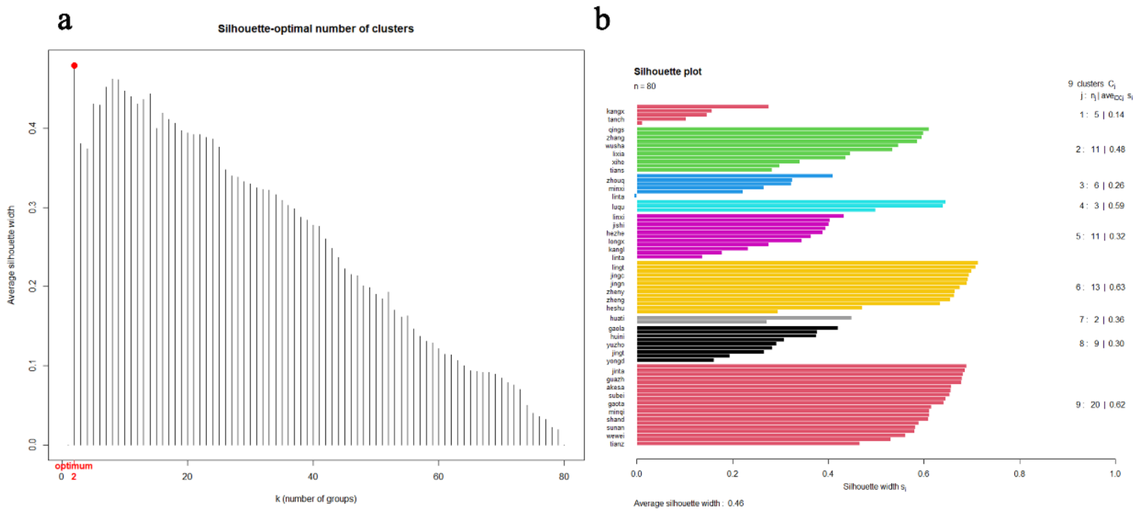

**Figure S1**. Plant phylogenetic flora fitting. Silhouette-optimal number of clusters k = 2 **(a)**. The cluster configuration is verified to be appropriate **(b)**. The silhouette value is a measure of how similar an object is to its own cluster (cohesion) compared to other clusters (separation). The silhouette ranges from −1 to +1, where a high value indicates that the object is well matched to its own cluster and poorly matched to neighboring clusters. If most objects have a high value, then the clustering configuration is appropriate. If many points have a low or negative value, then the clustering configuration may have too many or too few clusters.

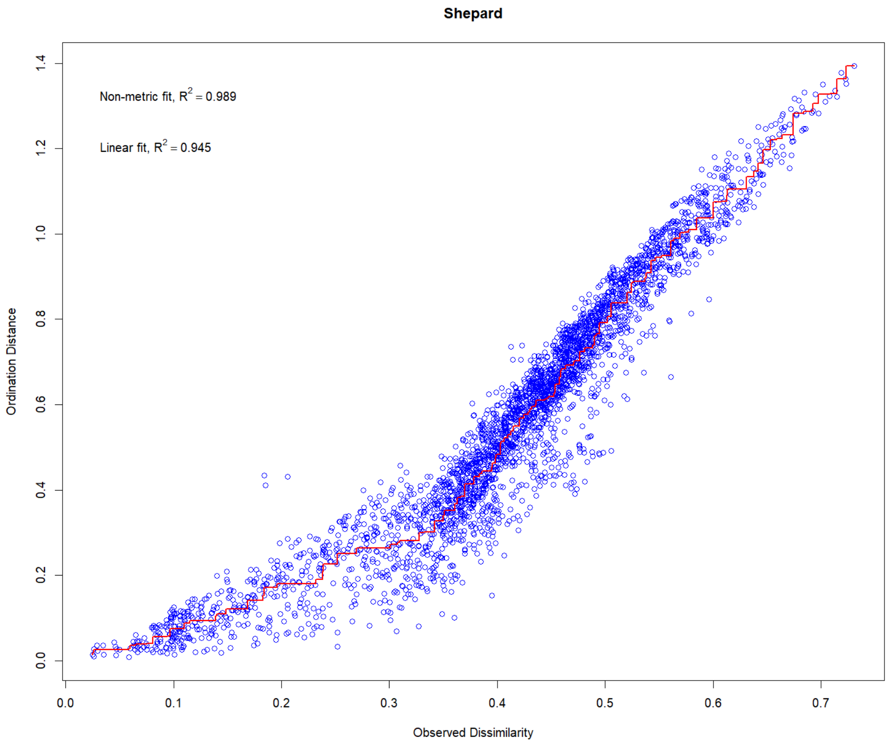


**Figure S2.** NMDS fitting plot. A higher fitting $R^2$ indicates a better fit.

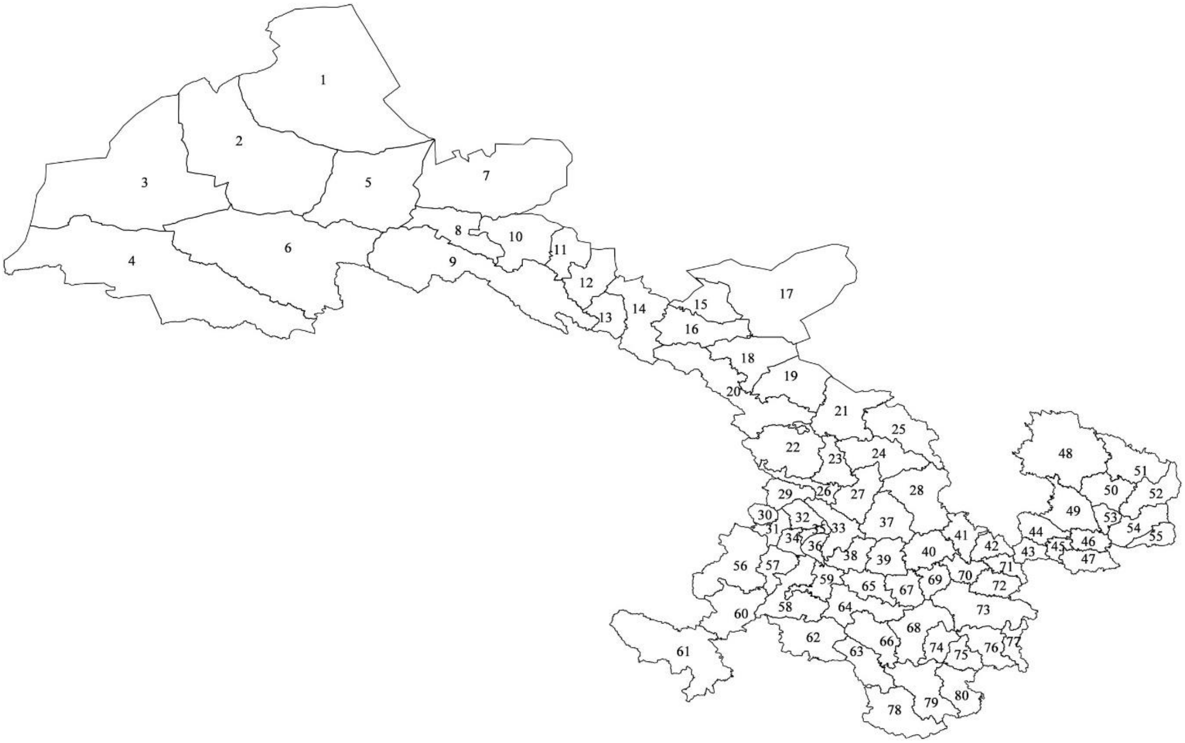


**Figure S3. Map of administrative counties in Gansu**. The numbers correspond with the code in Supplementary Table S1, which indicate name of each county. The map was generated using DIVA-GIS 10.8.

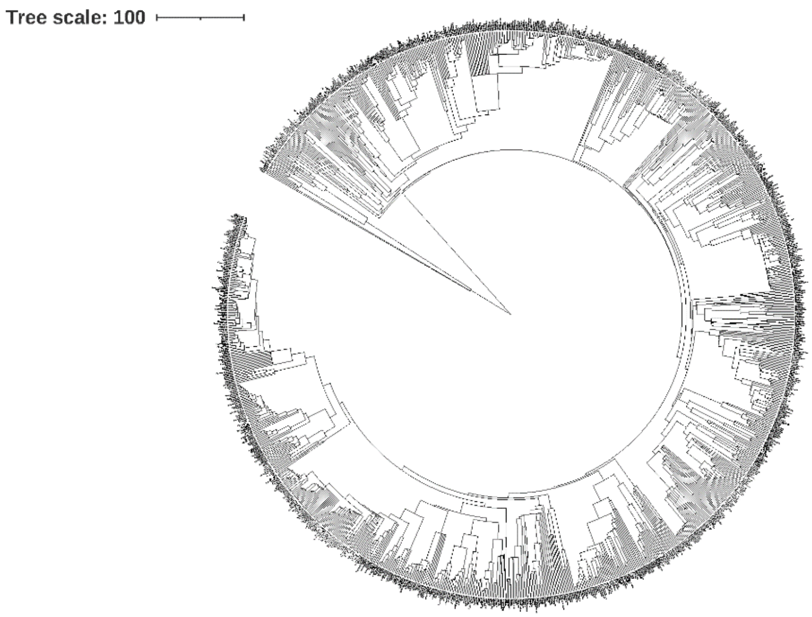


**Figure S4**. Phylogenetic tree of seed plant at the genus-level in Gansu.

## 2.2 Supplementary Tables

**Table S1. Area and floristic regions for each county in Gansu.** Code corresponds with the numbers in map of administrative counties (Supplementary Figure S1), which show the position of each county in Gansu.

| Code | County | Area(km$^2$) | Floristic region |
|---|---|---|---|
| 1 | North-Subei | 38000 | II |
| 2 | Guazhou | 24100 | II |
| 3 | Dunhuang | 31200 | II |
| 4 | Akesai | 32374 | I |
| 5 | Yumen | 13389 | II |
| 6 | South-Subei | 28748 | I |
| 7 | Jinta | 18800 | II |
| 8 | Suzhou | 6321 | II |
| 9 | Sunan | 14780 | I |
| 10 | Gaotai | 6016 | II |
| 11 | Linze | 2729 | II |
| 12 | Ganzhou | 4240 | II |
| 13 | Minle | 3687 | I |
| 14 | Shandan | 9374 | I |
| 15 | Yongchang | 5867 | II |
| 16 | Jinchuan | 3060 | II |
| 17 | Minqin | 15907 | II |
| 18 | Liangzhou | 5081 | II |
| 19 | Gulang | 5103 | I |
| 20 | Tianzhu | 7147 | I |
| 21 | Jingtai | 5483 | III |
| 22 | Yongdeng | 5652 | III |
| 23 | Gaolan | 2556 | III |
| 24 | Baiyin | 4534.2 | III |
| 25 | Jingyuan | 4754.8 | III |
| 26 | Lanzhou | 1630 | III |
| 27 | Yuzhong | 3245 | III |
| 28 | Huining | 6439 | III |
| 29 | Yongjing | 1864 | IV |
| 30 | Jishishan | 910 | IV |
| 31 | Linxia | 1302 | IV |
| 32 | Dongxiang | 1512 | IV |
| 33 | Lintao | 2851 | IV |
| 34 | Hezheng | 960 | IV |
| 35 | Guanghe | 538 | IV |
| 36 | Kangle | 1083 | IV |
| 37 | Anding | 4225 | III |
| 38 | Weiyuan | 2034 | IV |
| 39 | Longxi | 2657 | IV |
| 40 | Tongwei | 2899 | IV |
| 41 | Jingning | 2193 | V |

| | | | |
|---|---|---|---|
| 42 | Zhuanglang | 1558 | V |
| 43 | Huating | 1183 | IX |
| 44 | Kongtong | 1936 | IX |
| 45 | Chongxin | 852 | V |
| 46 | Jingchuan | 1409 | V |
| 47 | Lingtai | 2038 | V |
| 48 | Huanxian | 9236 | V |
| 49 | Zhenyuan | 3500 | V |
| 50 | Qingcheng | 2673 | V |
| 51 | Huachi | 3776 | V |
| 52 | Heshui | 2976 | V |
| 53 | Xifeng | 996 | V |
| 54 | Ningxian | 2633 | V |
| 55 | Zhengning | 1329 | V |
| 56 | Xiahe | 6266 | VIII |
| 57 | Hezuo | 2670 | VII |
| 58 | Zhuoni | 4920 | VII |
| 59 | Lintan | 2057 | VII |
| 60 | Luqu | 4260 | VIII |
| 61 | Maqu | 10190 | VIII |
| 62 | Diebu | 5148 | VII |
| 63 | Zhouqu | 3010 | VII |
| 64 | Minxian | 3500 | VII |
| 65 | Zhangxian | 2164 | VI |
| 66 | Tanchang | 3315 | IX |
| 67 | Wushan | 2011 | VI |
| 68 | Lixian | 4264 | VI |
| 69 | Gangu | 1573 | VI |
| 70 | Qinan | 1602 | VI |
| 71 | Zhangjiachuan | 1311 | VI |
| 72 | Qingshui | 2012 | VI |
| 73 | Tianshui | 5922 | VI |
| 74 | Xihe | 1862 | VI |
| 75 | Chengxian | 1678 | IX |
| 76 | Huixian | 2699 | VI |
| 77 | Liangdang | 1408 | VI |
| 78 | Wenxian | 5002 | IX |
| 79 | Wudu | 4642 | IX |
| 80 | Kangxian | 2968 | IX |
